# Bacterial genomes recovered from litter’s metagenomes in Amazonian Dark Earths

**DOI:** 10.1128/mra.00422-24

**Published:** 2024-06-04

**Authors:** Anderson Santos de Freitas, Luís Felipe Guandalin Zagatto, Gabriel Silvestre Rocha, Thierry Alexandre Pellegrinetti, Letícia de Cássia Malho Alves, Vitor Moreira de Lara, Jéssica Adriele Mandro, Guilherme Lucio Martins, Aleksander Westphal Muniz, Rogério Eiji Hanada, Luiz Fernando Würdig Roesch, Siu Mui Tsai

**Affiliations:** 1 Center for Nuclear Energy in Agriculture, University of Sao Paulo, Piracicaba, Brazil; 2 Department of Terrestrial Ecology, Netherlands Institute of Ecology, Wageningen, the Netherlands; 3 Department of Microbial Ecology, Netherlands Institute of Ecology, Wageningen, the Netherlands; 4 Western Amazon Agroforestry Research Center, Brazilian Agricultural Research Corporation, Manaus, Brazil; 5 General Coordination of Research, Training, and Extension, National Institute of Amazonian Research, Manaus, Brazil; 6 Department of Microbiology and Cell Science, University of Florida, Gainesville, Florida, USA; DOE Joint Genome Institute, Berkeley, California, USA

**Keywords:** next-generation sequencing, Amazon Rainforest, environmental DNA, genome assembly

## Abstract

Here, we report 27 metagenome-assembled bacterial genomes (MAGs) from litter samples of a secondary forest located in Brazil over an Amazonian Dark Earth pool. The data set includes members from the phyla Pseudomonadata (14 MAGs), Actinomycetota (7 MAGs), Bacteroidota (4 MAGs), Bacillota (1 MAG), and Bdellovibrionota (1 MAG).

## ANNOUNCEMENT

Forest litter is a crucial component for nutrient cycling, harboring diverse microbes with biotechnological potential (1). Amazonian Dark Earths are small patches of high-fertility soil in the Amazon that were enriched by the pre-Columbian native people who inhabited these areas and manipulated the soil with ceramic pieces, charcoal, and animal bones (2, 3). Here, we present 27 bacterial metagenome-assembled genomes (MAGs) obtained from litter samples from a secondary ADE forest, a potential hotspot for prospecting microbes that could enhance agricultural practices and ecological restoration (4, 5).

Six litter samples were collected on November 2021 from the Caldeirão Experimental Research Station, located in Iranduba-AM, Brazil (60°23′00″ W, 03°26′00″S). Each sample was obtained from distinct points (15 m apart from each) in a 40-year secondary forest with a horizon A/AB anthropic characterized as ADE using a 25 cm^2^ frame randomly placed on the forest. This study was registered in the Brazilian National System for Management of Genetic Patrimonial and Associated Traditional Knowledge (SISGEN) under the access number AD13FB3.

For each sample, 1 g of chopped litter was added into a 50 mL sterile tube filled with 40 mL of autoclaved Phosphate-Buffered Saline (PBS; 1×, pH 7.4) and incubated at 4°C with a shaking speed of 300 rpm for 12 h. The extracts were filtered through a 2 mm sieve to remove leaf particles to another sterile 50 mL tube and centrifuged at 12,000 × *g* for 15 min. The pellet was used for DNA extraction using the DNeasy PowerLyzer PowerSoil Kit (Qiagen, Hilden, Germany) following the recommendations for tropical soils (6). Libraries were prepared using NEBNext Ultra II DNA Library Prep Kit for Illumina (New England Biolabs, Ipswich, USA), and samples were sequenced using the shotgun approach at the NovaSeq 6000 platform (Illumina Inc., San Diego, USA) by the method paired-end 2 × 150 bp.

**Fig 1:**
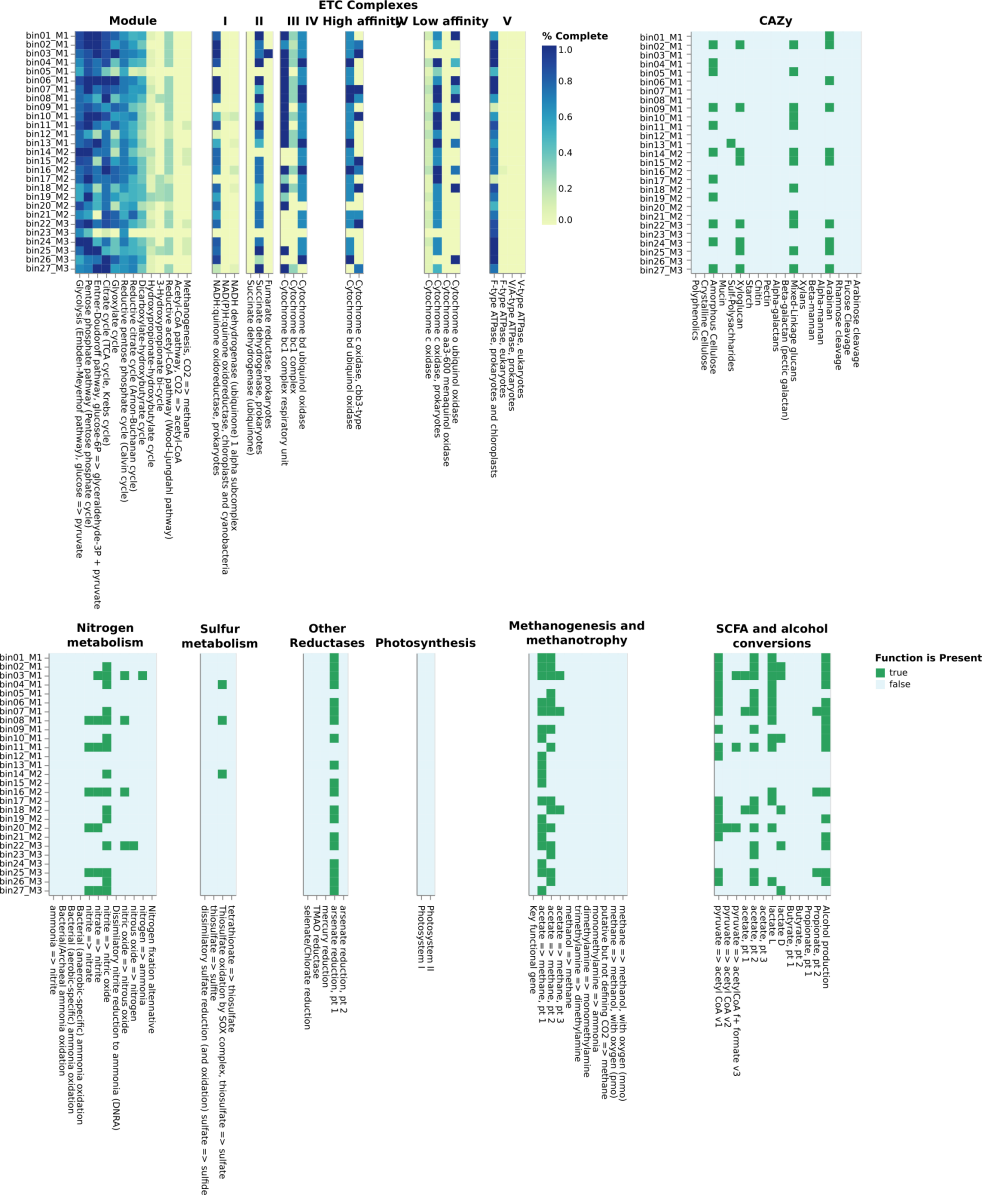
DRAM annotations of the 27 MAGs recovered from the metagenomes of litter in Amazonian Dark Earths.

Raw reads were processed in KBase (7). Default parameters were used for all software unless otherwise noted. Quality check was carried out using Trimmomatic v.0.36 (8). Three different methods were used to retrieve the MAGs. First, each sample was *de novo* assembled using metaSPAdes v3.15.5 assembler (9), evaluated with QUAST v5.2.0 (10), and bins were generated with MetaBAT 2 (11). Second, a co-assembly of the six samples (merged fastq files) was performed using metaSPAdes. Bins were retrieved using MetaBAT 2, MaxBin2 v2.2.4 (12), and Concoct v1.1 (13). The three binned results were added to DASTool v1.1.2 for refinement and de-replication (14). The third method was performed by importing the co-assembled reads into the COMEBin v1.0.3 (15). All bins generated in all methods were de-replicated using dRep v3.1.0 (16). De-replicated bins were quality-checked considering completeness and contamination (17) with CheckM v1.0.18 (18). The best quality MAGs (>70% completeness, <5% contamination) were then taxonomically annotated with GTDB-Tk v2.3.2 (19), and Prokka v1.14.5 (20), and functionally annotated with DRAM v0.1.2 (21), respectively (Table 1)(Fig. 1).

**TABLE 1:** Description of 27 MAGs obtained from litter samples in a secondary Amazonian Dark Earth forest^*a*^

MAG	GTDB classification	SRA identifier	GenBank identifier	Contigs	Completeness	Contamination	Total length	GC%	N50	CDS	rRNA	tRNA	tmRNA
bin01_M1	d__Bacteria;p__Pseudomonadota;c__ Gammaproteobacteria;o__Pseudomonadales; f__Cellvibrionaceae;g__Cellvibrio;s__	SAMN40501439	JBCECX000000000	74	97.04	0.38	5,128,080	47.47	128,266	4,306	0	28	1
bin02_M1	d__Bacteria;p__Pseudomonadota;c__ Gammaproteobacteria;o__Pseudomonadales; f__Pseudomonadaceae;g__Pseudomonas_E;s__	SAMN40501440	JBCECW000000000	653	76.94	0.82	4,102,145	62.12	7,014	3,894	0	25	0
bin03_M1	d__Bacteria;p__Pseudomonadota;c__ Gammaproteobacteria;o__Burkholderiales; f__Rhodocyclaceae;g__Dactylopiibacterium;s__	SAMN40501441	JBCECV000000 00	99	96.78	1.24	3,325,840	68.58	53,501	3,030	0	41	1
bin04_M1	d__Bacteria;p__Pseudomonadota;c__Gammaproteobacteria; o__Burkholderiales;f__Burkholderiaceae;g__Oxalicibacterium; s__Oxalicibacterium faecigallinarum	SAMN40501442	JBCECU000000000	164	95.59	0.43	2,979,859	54.97	25,452	2,906	0	38	1
bin05_M1	d__Bacteria;p__Actinomycetota;c__Actinomycetia; o__Actinomycetales;f__Microbacteriaceae;g__Curtobacterium; s__Curtobacterium sp900086645	SAMN40501443	JBCECT000000000	428	79.38	2.87	3,447,546	70.94	10,039	3,415	0	48	1
bin06_M1	d__Bacteria;p__Pseudomonadota;c__Gammaproteobacteria; o__Xanthomonadales;f__Rhodanobacteraceae;g__Luteibacter; s__Luteibacter sp000745055	SAMN40501444	JBCECS000000000	37	98.78	0.63	4,572,070	64.98	293,888	4,040	1	48	1
bin07_M1	d__Bacteria;p__Pseudomonadota;c__Gammaproteobacteria; o__Burkholderiales;f__Burkholderiaceae_B;g__ALPHA2B;s__	SAMN40501445	JBCECR000000000	672	98.98	0.82	5,438,842	69.19	10,528	5,030	0	42	0
bin08_M1	d__Bacteria;p__Pseudomonadota;c__Gammaproteobacteria; o__Burkholderiales;f__Burkholderiaceae_C;g__Bordetella; s__Bordetella sp002261335	SAMN40501446	JBCECQ000000000	199	90.52	0.62	5,097,444	70.34	41,725	4,827	1	42	1
bin09_M1	d__Bacteria;p__Actinomycetota;c__Actinomycetia; o__Actinomycetales;f__Microbacteriaceae;g__Leifsonia;s__	SAMN40501447	JBCECP000000000	71	89.32	0.88	5,023,944	58.24	221,190	4,662	0	41	1
bin10_M1	d__Bacteria;p__Pseudomonadota;c__Alphaproteobacteria; o__Rhizobiales;f__Rhizobiaceae;g__Agrobacterium; s__Agrobacterium cavarae	SAMN40501448	JBCECO000000000	180	97.3	0.87	3,634,223	70.85	35,746	3,617	0	39	1
bin11_M1	d__Bacteria;p__Actinomycetota;c__Actinomycetia; o__Actinomycetales;f__Cellulomonadaceae; g__Cellulomonas;s__	SAMN40501449	JBCECN000000000	181	95.17	0.0	1,609,008	35.46	12,035	1,429	0	24	1
bin12_M1	d__Bacteria;p__Pseudomonadota;c__Gammaproteobacteria; o__Diplorickettsiales;f__Diplorickettsiaceae;g__Rickettsiella_B;s__	SAMN40501450	JBCECM000000000	319	86.43	0.87	3,062,255	68.66	12,901	3,100	0	40	0
bin13_M1	d__Bacteria;p__Pseudomonadota;c__Alphaproteobacteria; o__Caulobacterales;f__Caulobacteraceae;g__Brevundimonas;s__	SAMN40501451	JBCECL000000000	139	87.69	1.93	3,434,383	72.37	38,335	3,111	0	47	0
bin14_M2	d__Bacteria;p__Bacteroidota;c__Bacteroidia;o__Sphingobacteriales; f__Sphingobacteriaceae;g__Mucilaginibacter;s__	SAMN40501452	JBCECK000000000	325	93.71	3.97	6,024,919	41.41	33,895	5,116	1	38	1
bin15_M2	d__Bacteria;p__Bacteroidota;c__Bacteroidia;o__Flavobacteriales; f__Flavobacteriaceae;g__Flavobacterium;s__Flavobacterium lindanitolerans	SAMN40501453	JBCECJ000000000	221	96.37	0.35	3,626,744	37.68	29,068	3,211	0	29	1
bin16_M2	d__Bacteria;p__Pseudomonadota;c__Alphaproteobacteria; o__Rhizobiales;f__Rhizobiaceae;g__Ochrobactrum_A; s__Ochrobactrum_A pseudogrignonensis	SAMN40501454	JBCECI000000000	344	92.05	2.83	4,239,545	53.72	18,488	4,060	0	38	1
bin17_M2	d__Bacteria;p__Actinomycetota;c__Actinomycetia;o__Actinomycetales; f__Microbacteriaceae;g__Microbacterium;s__	SAMN40501455	JBCECH000000000	342	83.51	1.73	3,382,307	69.93	15,560	3,286	1	34	0
bin18_M2	d__Bacteria;p__Pseudomonadota;c__Alphaproteobacteria; o__Acetobacterales;f__Acetobacteraceae; g__Paracraurococcus;s__Paracraurococcus sp013112485	SAMN40501456	JBCECG000000000	817	81.88	4.27	4,547,200	72.04	6,566	4,322	1	43	1
bin19_M2	d__Bacteria;p__Actinomycetota;c__Thermoleophilia; o__Solirubrobacterales;f__Solirubrobacteraceae; g__JAGIBJ01;s__	SAMN40501457	JBCECF000000000	675	86.37	0.98	4,429,271	71.80	8,477	4,226	1	47	1
bin20_M2	d__Bacteria;p__Actinomycetota;c__Actinomycetia; o__Propionibacteriales;f__Propionibacteriaceae; g__Propionicimonas;s__	SAMN40501458	JBCECE000000000	637	74.89	0.69	3,163,046	71.50	5,539	3,188	0	61	1
bin21_M2	d__Bacteria;p__Bdellovibrionota;c__Bdellovibrionia; o__Bdellovibrionales;f__Bdellovibrionaceae;g__Bdellovibrio;s__	SAMN40501459	JBCECD000000000	473	89.66	0.51	3,024,043	46.47	7,644	3,038	1	30	0
bin22_M3	d__Bacteria;p__Bacteroidota;c__Bacteroidia; o__Flavobacteriales;f__Flavobacteriaceae;g__Flavobacterium; s__Flavobacterium nitrogenifigens	SAMN40501460	JBCECC000000000	148	97.45	1.43	5,295,192	33.77	70,500	4,594	1	43	1
bin23_M3	d__Bacteria;p__Bacillota;c__Bacilli;o__Mycoplasmatales; f__VBWQ01;g__Spiroplasma_D;s__	SAMN40501461	JBCECB000000000	8	95.49	0.75	807,964	26.76	184,592	1,405	3	27	1
bin24_M3	d__Bacteria;p__Bacteroidota;c__Bacteroidia; o__Sphingobacteriales;f__Sphingobacteriaceae;g__Pedobacter;s__	SAMN40501462	JBCECA000000000	27	97.13	0.0	3,903,772	43.98	297,924	4,362	2	32	1
bin25_M3	d__Bacteria;p__Actinomycetota;c__Actinomycetia; o__Mycobacteriales;f__Mycobacteriaceae;g__Rhodococcus;s__	SAMN40501463	JBCEBZ000000000	597	89.51	3.24	7,655,958	68.18	17,547	7,051	0	47	1
bin26_M3	d__Bacteria;p__Pseudomonadota;c__Gammaproteobacteria; o__Pseudomonadales;f__Moraxellaceae;g__Acinetobacter; s__Acinetobacter pittii	SAMN40501464	JBCEBY000000000	569	87.0	1.15	3,290,807	38.92	6,445	3,075	0	31	1
bin27_M3	d__Bacteria;p__Pseudomonadota;c__Alphaproteobacteria; o__Caulobacterales;f__Caulobacteraceae;g__Asticcacaulis;s__	SAMN40501465	JBCEBX000000000	394	82.8	1.73	2,719,478	56.58	8,186	2,687	0	31	0

^*a*^ Protein coding sequence.

Our data provide a better understanding of the bacterial roles in nutrient cycling within ADE litter. We expect these results to be useful for future research in soil biotechnology.

## Data Availability

This Whole Genome Shotgun project has been deposited in GenBank under accession no. PRJNA1088826. The raw reads can be found in SRA under the accession numbers SRR25306242, SRR25306243, SRR25306244, SRR25306239, SRR25306240, and SRR25306241. The average number of reads per sample was 40.1 ± 7.1 million reads. The version described in this paper is the first version. The annotation files can be found on GitHub at the address https://github.com/FreitasAndy/LitterMAGS.
